# SGLT2 Inhibitor, Canagliflozin, Attenuates Myocardial Infarction in the Diabetic and Nondiabetic Heart

**DOI:** 10.1016/j.jacbts.2018.10.002

**Published:** 2019-01-30

**Authors:** Ven G. Lim, Robert M. Bell, Sapna Arjun, Maria Kolatsi-Joannou, David A. Long, Derek M. Yellon

**Affiliations:** a The Hatter Cardiovascular Institute, University College London, London, United Kingdom; b Developmental Biology and Cancer Programme, UCL Great Ormond Street Institute of Child Health, London, United Kingdom

**Keywords:** cardioprotection, diabetes, ischemia-reperfusion injury, myocardial infarction, SGLT2 inhibitor, DMSO, dimethyl sulfoxide, NHE, sodium hydrogen exchange, NS, not significant, SGLT2, sodium glucose transporter 2, ZDF, Zucker Diabetic Fatty, ZL, Zucker Lean

## Abstract

The authors hypothesized that despite similar cardiovascular event rates, the improved cardiovascular survival from sodium glucose transporter 2 (SGLT2) inhibition, seen clinically, could be via a direct cytoprotective effect, including protection against myocardial ischemia/reperfusion injury. Langendorff-perfused hearts, from diabetic and nondiabetic rats, fed long-term for 4 weeks with canagliflozin, had lower infarct sizes; this being the first demonstration of canagliflozin’s cardioprotective effect against ischemia/reperfusion injury in both diabetic and nondiabetic animals. By contrast, direct treatment of isolated nondiabetic rat hearts with canagliflozin, solubilized in the isolated Langendorff perfusion buffer, had no impact on infarct size. This latter study demonstrates that the infarct-sparing effect of long-term treatment with canagliflozin results from either a glucose-independent effect or up-regulation of cardiac prosurvival pathways. These results further suggest that SGLT2 inhibitors could be repurposed as novel cardioprotective interventions in high-risk cardiovascular patients irrespective of diabetic status.

## Highlights

• Long-term SGLT2 inhibition with dietary canagliflozinin diabetic *and* nondiabetic rats attenuates myocardial ischemia/reperfusion injury ex vivo.
• This suggests that the improvement in myocardial infarct size by SGLT2 inhibition may occur independent of the glycemic status.
• Canagliflozin improved hyperglycemia in diabetic rats but importantly did not cause hypoglycemia in nondiabetic rats.
• Short-term perfusion of the nondiabetic heart with canagliflozin, solubilized in the Langendorff perfusion buffer, had no impact on the myocardial infarct size.

## Visual Abstract

**Figure:**
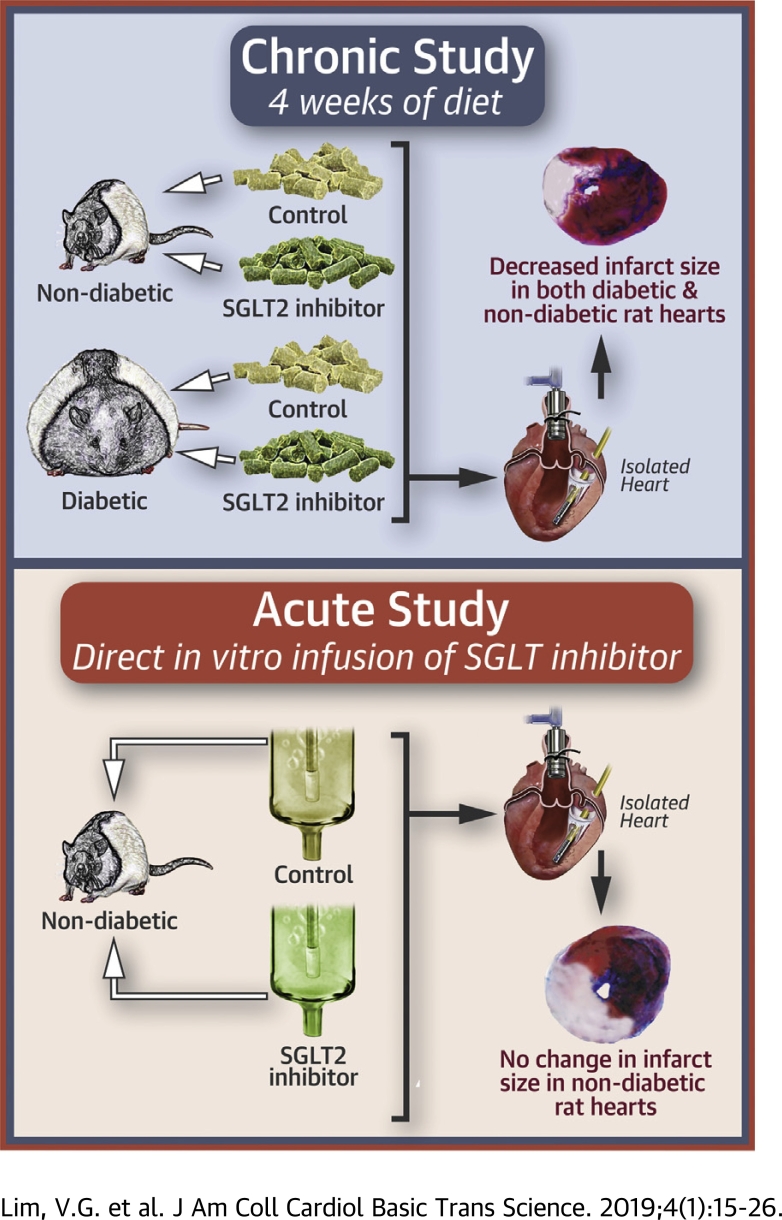

The remarkable cardiovascular benefits of sodium/glucose co-transporter 2 (SGLT2) inhibitors are now well recognized in high-risk type 2 diabetic patients following the landmark clinical trials, EMPA-REG OUTCOME (Empagliflozin Cardiovascular Outcome Event Trial in Type 2 Diabetes Mellitus) (1) and CANVAS (CANagliflozin CardioVAScular Assessment Study) (2), and is further supported by positive outcome data from DECLARE-TIMI 58 trial (Multicenter Trial to Evaluate the Effect of Dapagliflozin on the Incidence of Cardiovascular Events-Thrombolysis In Myocardial Infarction 58) announced at the recent European Society of Cardiology World Congress. These studies, both designed as noninferiority investigations mandated by the regulatory authorities, revealed an unexpected benefit and superiority over existing standard diabetic care, with a significant reduction of cardiovascular mortality. Equally remarkably, this reduction in cardiovascular mortality was seen notably early—within 1 to 2 months—following the introduction of the respective SGLT2 inhibitor. The mechanism underlying the reduction in cardiovascular mortality is not clear and has been subject to much conjecture: seemingly, improvements in blood sugar control were comparatively minor and improvements in terms of diuresis, weight loss, and blood pressure reduction inadequate to fully explain the differences observed. Indeed, many, including ourselves, have speculated a potential pleiotropic beneficial effect for this class of glucose-lowering therapy 3, 4, 5.

The hypothesis that SGLT2 inhibitors may have pleiotropic effects appears to be supported by other observations from the clinical trial data, not least that SGLT2 inhibition appears to have minimal impact upon the cardiovascular event rate—be it myocardial infarction or stroke, admissions with unstable angina or the need for a coronary revascularization procedure 1, 2. As such, there appears to be minimal impact of SGLT2 inhibition upon macrovascular (arterial atheromatous) disease—but overall, despite experiencing the same frequency of cardiovascular events, survival nonetheless appears to be better in those taking SGLT2 inhibitors, a benefit that strikingly manifests within the first few months of treatment.

Cellular injury, necrosis, and programmed cell death (apoptosis, necroptosis, autophagy) are important pathophysiological features of a number of maladaptive processes in the heart, including myocardial ischemia and heart failure (6). We therefore hypothesized that despite a similar cardiovascular event rate from events such as acute myocardial ischemia, the improved cardiovascular survival arising from SGLT2 inhibition was through direct myocardial cytoprotection. In a rat, this can be tested in an experimental model of injurious ischemia/reperfusion injury, whereby diabetic animals treated with an SGLT2 inhibitor would be anticipated to have smaller myocardial infarcts. Moreover, if the cardiovascular benefits of SGLT inhibitors are genuinely pleiotropic, we hypothesized that the benefits of SGLT2 inhibition would also be found in those without diabetes.

In designing our experiments, we observed that whereas the survival curves in the EMPA-REG and CANVAS trials separate quickly, it still takes weeks to see the survival curves diverge. As such, we undertook to treat both diabetic and nondiabetic rats for a period of 4 weeks. Moreover, because treatment with an SGLT2 inhibitor will invariably affect circulating blood glucose at the time of myocardial infarction in vivo, we harvested the hearts and undertook the experiments in an ex vivo Langendorff model, with perfused glucose concentration controlled in all experiments.

Finally, we wished to ascertain whether the SGLT2 inhibitor would have a direct, cardioprotective effect in the isolated heart, and to this end, we undertook a further group of experiments with “acute” exposure to the SGLT2 inhibitor, with the drug added to the Langendorff perfusate throughout the perfusion protocol.

Using the SGLT2 inhibitor, canagliflozin, in a reverse-translational study, we found that long-term pre-administration over 4 weeks led to a significant attenuation of myocardial infarct size in both diabetic Zucker Diabetic Fatty (ZDF) and nondiabetic Zucker Lean (ZL) rats. This observation may have significant impact for future translational studies in the repurposing of this new class of glucose-lowering drugs in all patients, irrespective of diabetic status, with high-risk cardiovascular disease.

## Methods

For a detailed description of all methods, see the Supplemental Appendix. In brief, ZL and ZDF rats were monitored weekly with random blood glucose assessment, and fed either standard or high-fat chow, either fortified with canagliflozin or without (control) for a period of 4 weeks before harvesting the heart and Langendorff perfusion. All feeds, both with and without drug, were prepared by Research Diets (New Brunswick, New Jersey) based on the diet formulations provided by Janssen Research and Development (Springhouse, Pennsylvania). Using this formulation, the canagliflozin-fortified feed results in a circulating canagliflozin concentration (10 μmol/l) equivalent to that found in human subjects taking maintenance canagliflozin, 300 mg daily (7). Different diets were used for nondiabetic ZL and diabetic ZDF rats to account for the quantity of food eaten by these rats: the details of these feeds are detailed in the Supplemental Appendix.

Animals used for the acute administration of canagliflozin were nondiabetic Sprague-Dawley rats where canagliflozin (Janssen Research and Development) or vehicle, dimethyl sulfoxide (DMSO) (0.05% DMSO, Sigma Aldrich, Poole, United Kingdom) was perfused throughout the Langendorff experiment.

### Randomization

All experiments were block randomized. Analysis was performed by 2 blind observers and arbitrated by a third independent adjudicator if required. Once all results were available, the data were unblinded and analyzed.

### Statistical analysis

All analyses were performed using GraphPad Prism version 6 (GraphPad Software, San Diego, California). The specific statistical test used is reported next to each result. An unpaired *t*-test was used for 2 independent groups of continuous variables and a 1-way analysis of variance with Tukey’s multiple comparison test for 3 or more independent groups. Data are presented as mean ± SEM. N values are either displayed in the figure or described in the figure legend for each experiment. A significance level of 5% (α = 0.05) and 80% power (β = 0.20) were used. Statistical significance was reported if p was <0.05 and results where p was >0.05 were reported as nonsignificant.

## Results

### Characterization of the ZDF diabetic phenotype

**Figure 1:**
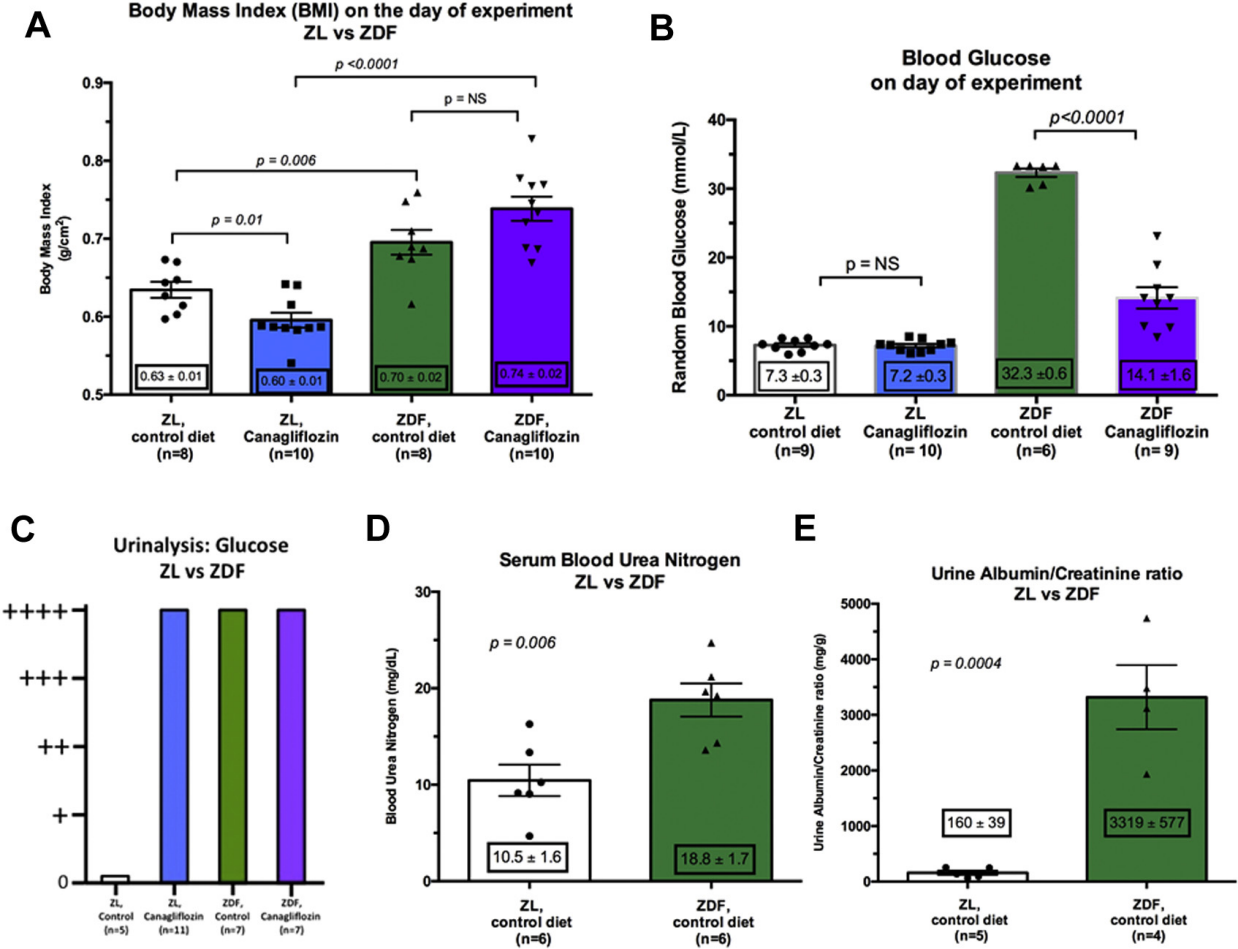
Characterization of the ZL and ZDF Phenotypes **(A)** Body weight index. The diabetic ZDF rats were significantly larger than the nondiabetic ZL rats. Canagliflozin administration in the ZL led to a significant reduction in body mass index that was absent in the ZDF diabetic rats. n = 8 to 10 per group. **(B)** Random glucose concentration on day of experiment. As expected, ZDF diabetic rats had significantly higher blood glucose concentrations compared to the nondiabetic ZL controls (p < 0.0001; n = 6 to 9 per group). Canagliflozin had no impact upon blood glucose in the ZL group (p = NS; n = 9 to 10 per group), but significantly reduced glucose in the diabetic ZDF rats (p < 0.0001; n = 6 to 9 per group). **(C to E)** Renal manifestations of diabetes in the ZDF rats. **(C)** Urine glucose, measured by urinalysis strip test. No glucosuria was detectable in the control ZL rats, but there was significant glucosuria in ZL rats on canagliflozin. As expected, significant glucosuria was found in both ZDF control and canagliflozin-treated groups. **(D)** Blood urea nitrogen was significantly higher in the ZDF rats compared with the nondiabetic ZL: 11 ± 2 mg/dl versus 19 ± 2 mg/dl (p = 0.006, n = 6 per group). **(E)** A similar pattern was observed in the urine albumin/creatinine ratio—the diabetic ZDF rats demonstrating a significantly higher albumin excretion compared with the nondiabetic ZL rat: 160 ± 39 mg/g versus 3,319 ± 577 mg/g (p = 0.0004; n = 4 to 5 per group). ZDF = Zucker Diabetic Fatty; ZL = Zucker Lean.

To ensure that our ZDF rats represented a reasonable facsimile of the diabetic cohort represented within the EMPA-REG and CANVAS studies, we undertook characterization of the nondiabetic ZL and diabetic ZDF rats. We found, as expected, that the ZDF rats were obese and hyperglycemic (Figures 1A and 1B) and hyperglucosuric (Figure 1C). In addition, the ZDF rats were found to have evidence of end-organ manifestations of their diabetes, as represented by abnormal renal function and albuminuria (Figures 1C and 1D). We are therefore confident that the ZDF represents a reasonable approximation of the human obese type 2 diabetic phenotype with significant and established diabetes at the time of experimentation.

Unexpectedly, we found that diabetic rats treated with canagliflozin were heavier than untreated diabetic rats; the expected weight loss from the calorific depletion associated with SGLT2-dependent glycosuria was, however, observed in the canagliflozin-treated nondiabetic ZL rats. Growth curves are shown in Supplemental Figure 1: the control-diet diabetic ZDF rats started heavier than the nondiabetic ZL rats, but failed to gain significant weight over the 4 weeks of feeding. By contrast, nondiabetic ZL rats gained weight in a linear fashion over the same 4-week period. Interestingly, the pattern and rate of weight gain seen in nondiabetic rats were mirrored in diabetic ZDF rats fed with canagliflozin, suggesting a healthier animal concomitant with better-controlled diabetes, an interpretation fitting with empirical observations of these animals’ physical condition.

### Characterization of the efficacy of canagliflozin in lowering circulating glucose

**Figure 2:**
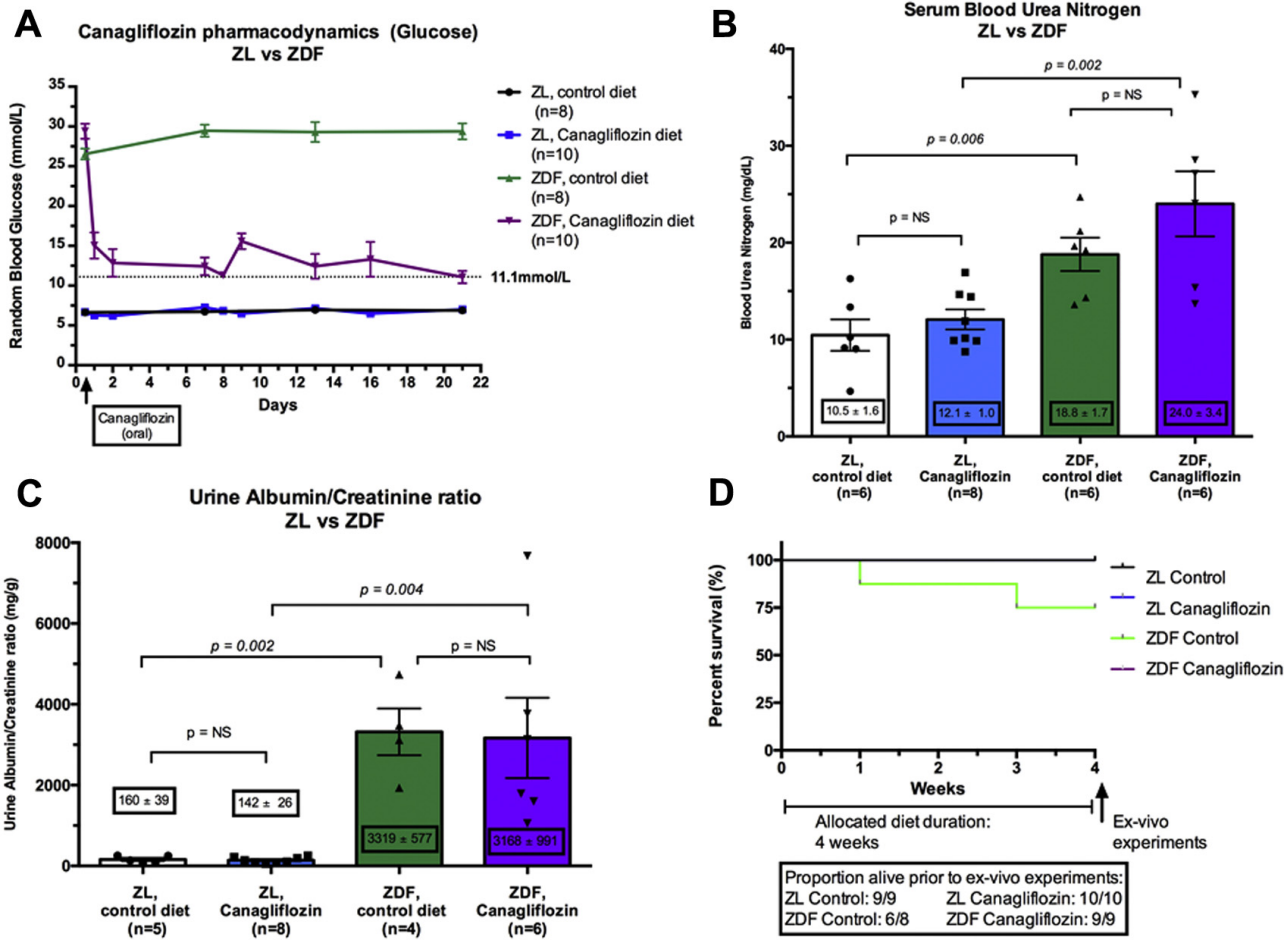
Impact of Canagliflozin in Nondiabetic ZL and Diabetic ZDF Rats **(A)** Canagliflozin had a rapid and sustained impact upon circulating blood glucose in the diabetic ZDF rats compared with the untreated animals. By contrast, canagliflozin had no impact upon circulating blood glucose in the nondiabetic ZL rats (p = 0.002; n = 8 to 10 per group). **(B)** After 4 week’s treatment, canagliflozin had little impact upon blood urea nitrogen in either ZL or ZDF rats (p = NS; n = 6 to 8 per group). **(C)** As with BUN, there was little impact from 4-week oral canagliflozin administration in either ZL or ZDF rats upon albumin/creatinine ratios (p = NS; n = 4 to 8 per group). **(D)** Kaplan-Meier survival curve. Two animals, both in the control diabetic ZDF group, had to be euthanized for severe urinary sepsis. All other groups completed without events. Abbreviations as in Figure 1.

To ensure that oral administration of canagliflozin, via fortification of the chow, was an effective antihyperglycemic intervention in our rat model, we observed the random glucose profile in both nondiabetic ZL and diabetic ZDF rats throughout the treatment lead-in period. We found that canagliflozin was highly effective in lowering blood glucose concentrations in the ZDF rats within a short period from the onset of oral drug administration. Significantly improved blood glucose control was evident throughout the canagliflozin treatment course compared with control, with random blood glucose of 16 ± 4 mmol/l versus 29 ± 1 mmol/l, respectively (p = 0.002) (Figure 2A).

Importantly, canagliflozin had no impact upon circulating glucose in the nondiabetic ZL rats, with equivalent blood glucose being recorded in both groups (p = NS) (Figure 2A). Importantly, we found no evidence of hypoglycemia in either canagliflozin treatment group, despite the presence of significant glucosuria in the canagliflozin-treated nondiabetic ZL rats (Figure 1C).

**Figure 3:**
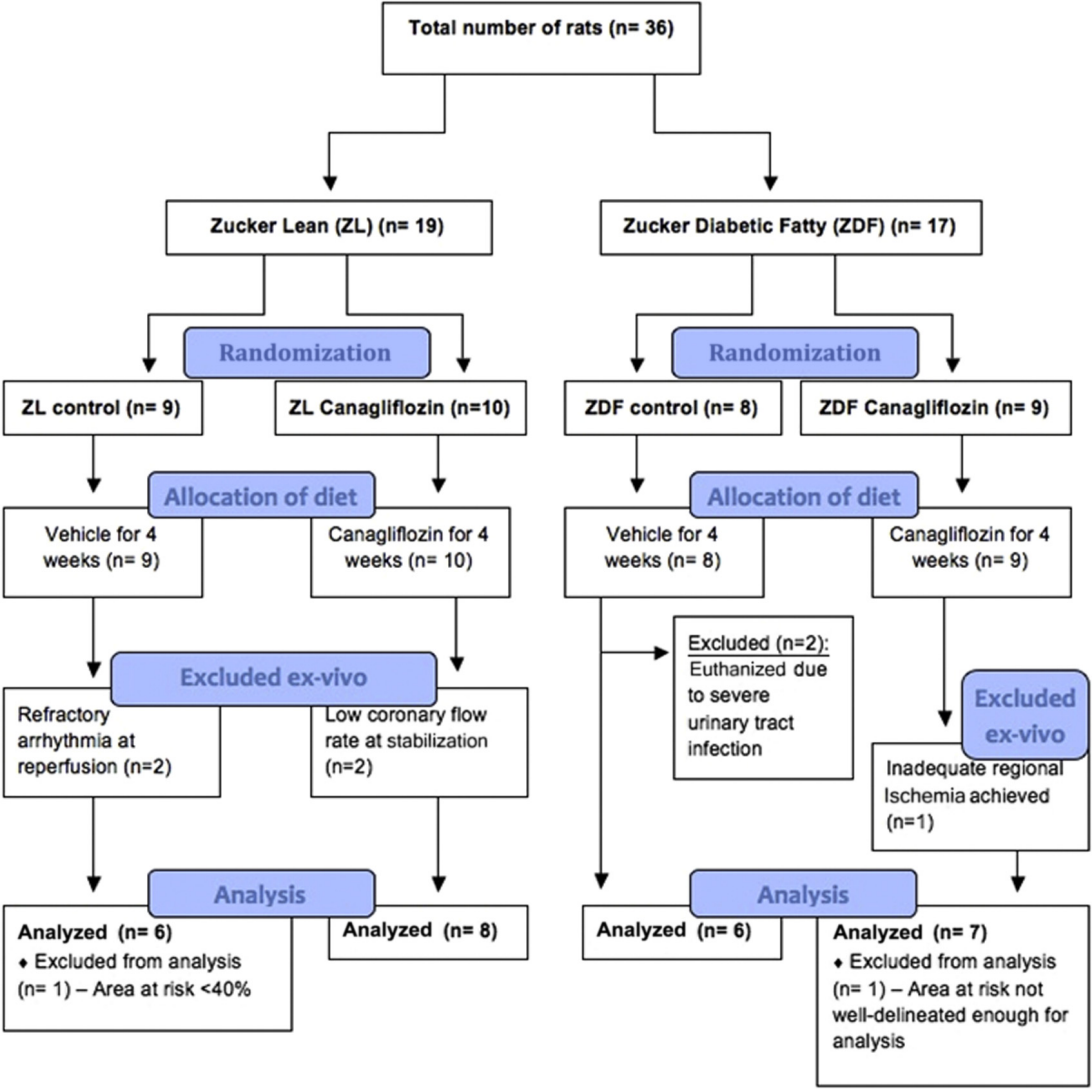
A CONSORT-Style Diagram for Infarct Assessment in the 4-Week Administration Study Thirty-six animals were started into the study, of which 29 completed through to analysis. Reasons for and timings of animal exclusions shown in all groups. Pre priori exclusion criteria are shown in the Supplemental Appendix. Abbreviations as in Figure 1.

Interestingly, there was no attenuation of renal dysfunction in the diabetic canagliflozin-treated group (p = NS) (Figures 2B and 2C). Unfortunately, our urinalysis assay saturates at glucose levels in excess of 110 mmol/l, but higher urinary glucose would be anticipated in this group (Figure 1C). With respect to animal mortality, only 2 deaths were recorded—both animals were euthanized for severe urinary tract infection, and these events were found to occur only in animals in the untreated control diabetic ZDF group (Figures 2D and 3). The impact of diabetes and canagliflozin upon un-paced heart rate and liver: body weight ratio are summarized in Supplemental Figures 3 and 4, respectively.

### Impact of 4-week oral canagliflozin on myocardial infarct size

For this investigation, we used 36 animals. Of these, 9 had to be excluded for reasons summarized in Figure 3. Twenty-seven animals completed the full experimental protocol.

**Figure 4:**
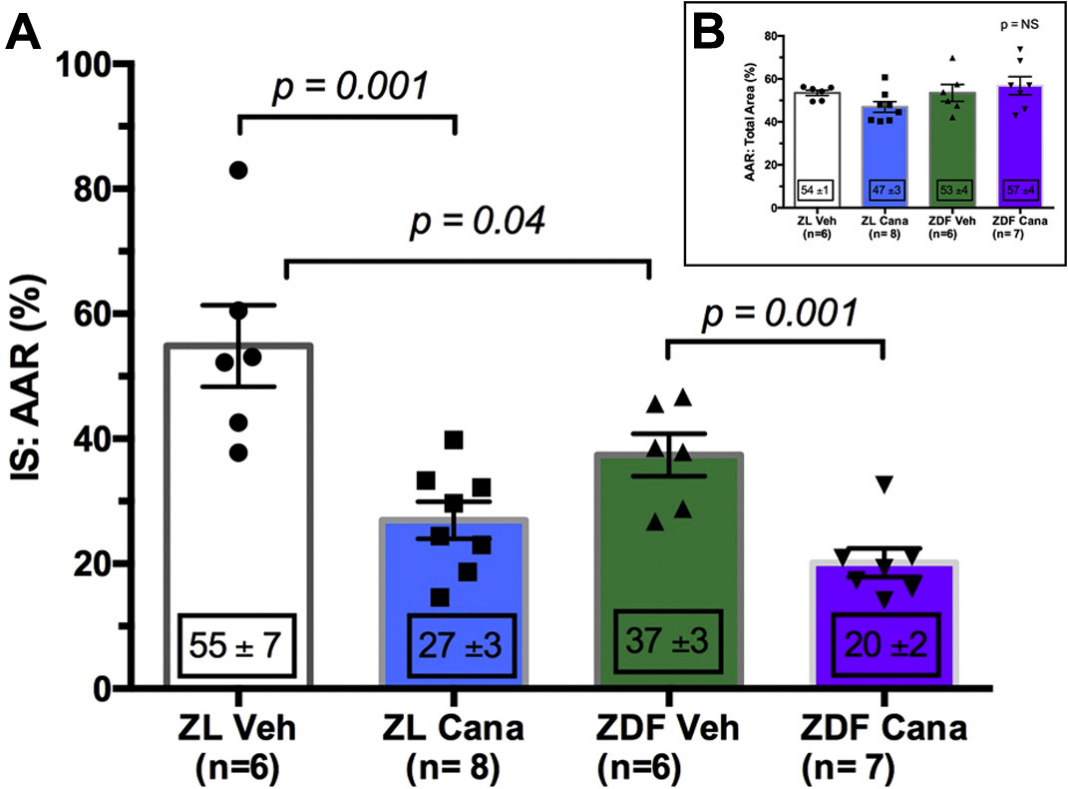
Infarct Size Reduction Following Long-Term 4-Week Oral Administration of Canagliflozin **(A)** In both diabetic ZDF and nondiabetic ZL rats, we found a significant reduction of infarct size compared with control. In nondiabetic rats, infarct size was reduced from 55 ± 7% to 27 ± 3% (p = 0.001; n = 6 to 8 per group). In the diabetic ZDF rats, a similar reduction of infarct size was also observed with infarct size reducing from 37 ± 3% to 20 ± 2% (p = 0.001; n = 6 to 8). There was a modest, but significant, difference in infarct size between control diet–treated ZL and ZDF rats (p = 0.04). **(B)** Area at risk in all groups was equivalent (p = NS; n = 6 to 8 per group). Cana = canagliflozin; IS:AAR = infarct size/area at risk ratio; ND = nonsignificant; Veh = vehicle; other abbreviations as in Figure 1.

We found a small, but significant, difference between myocardial infarct size in the control arms of the diabetic ZDF and the nondiabetic ZL rat heart groups (p = 0.04) (Figure 4A, Supplemental Figure 2). This difference is expected in Langendorff-perfused hearts where glucose is the sole energy substrate (see review [8]). We found that canagliflozin, mirroring the important data by Andreadou et al. (9) in the mouse, significantly reduced myocardial infarct size in diabetic ZDF rats. Infarct size relative to the control chow–fed ZDF rats was significantly attenuated, from 37 ± 3% to 20 ± 2% of the area at risk (p = 0.001) (Figure 4A). Importantly, canagliflozin also significantly abrogated myocardial injury in the nondiabetic ZL rats, reducing infarct size from 55 ± 7% to 27 ± 3% (p = 0.001) (Figure 4A). The areas at risk in all control and treatment groups were similar with no statistical difference (Figure 4B). The impact of canagliflozin upon coronary flow and left ventricular developed pressure are summarized in Supplemental Figures 5 and 6, respectively.

### Effect of short-term administration of canagliflozin at the time of ischemia/reperfusion injury

To ascertain whether acute administration of canagliflozin is protective against injurious ischemia/reperfusion injury in the nondiabetic rat, we subjected the isolated Sprague-Dawley rat heart to ischemia/reperfusion injury in the presence of vehicle (0.05% DMSO) or 10 μmol/l canagliflozin throughout the perfusion protocol (during 40-min stabilization, 35-min regional ischemia, and throughout the 2 h of reperfusion). The concentration used is equivalent to the plasma concentration of canagliflozin in diet-fed ZDF rats (7). Baseline characteristics were identical between groups: both demonstrating a nondiabetic level of random blood glucose and identical anthropological measurements between groups (Supplemental Table 1). No rats had to be excluded from this study, and all rat data were included in the final analysis.

**Figure 5:**
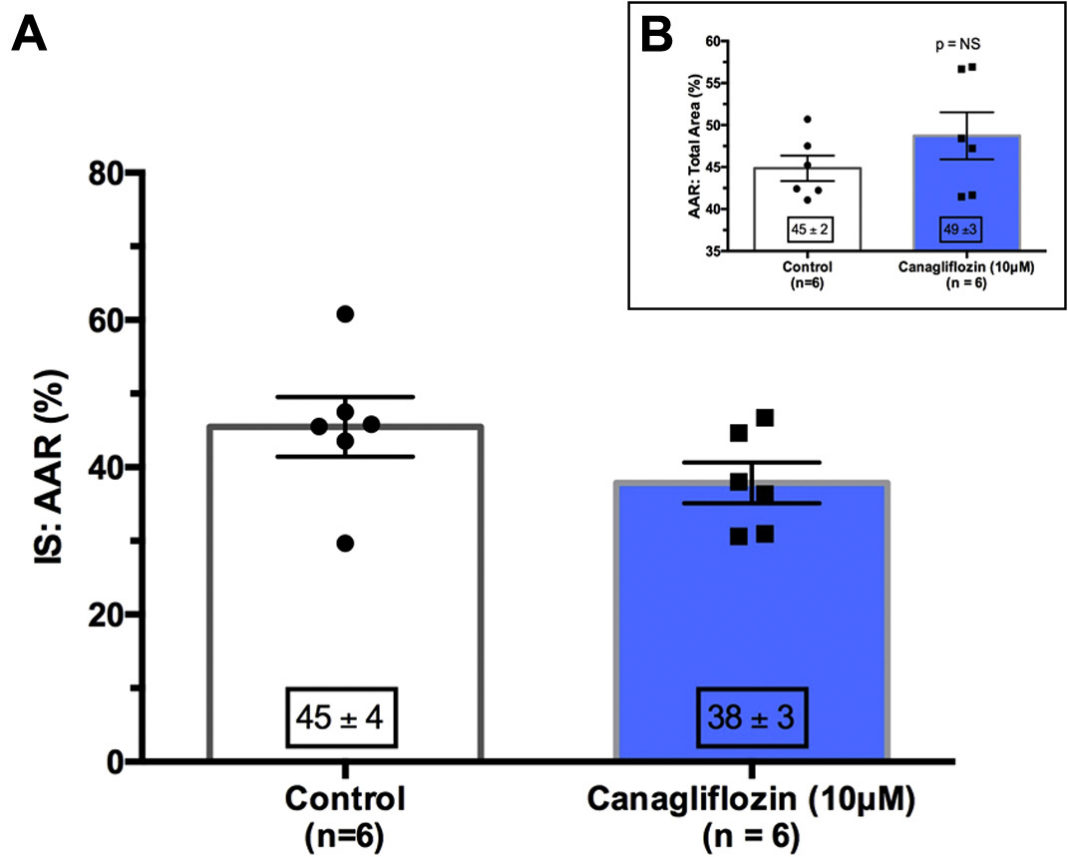
Infarct Sizes Following Short-Term Ex-Vivo Administration in Nondiabetic Sprague-Dawley Rats **(A)** In contrast to the cardioprotective effect of 4-week oral administration of canagliflozin, we found no evidence of infarct reduction with short-term, ex-vivo administration of canagliflozin: infarct sizes of 45 ± 4% versus 38 ± 3% (p = NS, n = 6 per group) in the vehicle control group. **(B)** There was no difference in the area at risk in either of the treatment groups (DMSO vehicle control versus canagliflozin; p = NS; n = 6 per group). DMSO = dimethyl sulfoxide; other abbreviations as in Figures 1 and 4.

Of note, short-term, ex-vivo canagliflozin failed to significantly alter infarct size, with treatment versus control of 38 ± 3% versus 45 ± 4%, respectively (p = 0.15) (Figure 5A). There was no difference in the area at risk between any of the groups (Figure 5B).

## Discussion

Our study provides the first evidence to our knowledge that long-term oral administration of canagliflozin over a period of 4 weeks is cardioprotective, ameliorating myocardial infarct size in both diabetic *and* nondiabetic rats, independent of glucose concentration at the time of ischemia/reperfusion injury. The latter observation, that canagliflozin-induced protection in the nondiabetic rat, is particularly noteworthy: a clinically available SGLT2 inhibitor, canagliflozin, appears to have a cardiovascular and cardioprotective role that extends beyond (and probably also independent of) its intended indication in the management of hyperglycemia in type 2 diabetes mellitus.

### Long-term oral canagliflozin attenuates myocardial infarction in the diabetic rat

In the diabetic ZDF rats, attenuating the extent of myocardial necrosis hints towards a novel mechanism underlying the significant reduction of cardiovascular mortality found in the clinical outcome studies, EMPA-REG and CANVAS 1, 2. Although the clinical data reveal no evidence that SGLT2 inhibitors reduce the number of cardiovascular events such as acute coronary syndromes, they may reduce the myocardial injury that occurs as a consequence of these events. A reduction of myocardial necrosis may thus improve both the immediate and long-term survivability of acute myocardial infarction and reduce the progression into ischemic cardiomyopathy and heart failure—a hypothesis that warrants further investigation.

Interestingly, the protection from long-term ingestion of canagliflozin was found in hearts that were removed and perfused, ex vivo, with a perfusate that contained a fixed concentration of glucose (11 mmol/l). We designed the experiments this way intentionally to avoid potential confounding the effects of glucose-lowering by canagliflozin at the time of ischemia/reperfusion injury. Moreover, Langendorff perfusion removes, through washout, other metabolic substrates that may confound canagliflozin administration (e.g., hepatic generation of ketones (10), as discussed further later in the text) are excluded as a potential mechanism of cardioprotection. Moreover, that these explanted hearts were protected, despite 40 min of crystalloid washout before ischemia, suggests a mechanism that imbues a “memory,” potentially through the recruitment of signaling pathways. And if a signaling pathway, it is a pathway whose efficacy, unlike that of ischemic conditioning (11), is seemingly not affected by the presence of significant diabetes (the severity of the diabetic phenotype confirmed by evidence of the development of nephropathy). One such mechanism may be through a Jak-STAT3 pathway, as suggested by Iliodromitis’s group (9)—but there may be others.

### Long-term oral canagliflozin attenuates myocardial infarction in the nondiabetic rat

Although the observation that canagliflozin attenuates infarct size in the diabetic rat is important, the principal novelty in this study comes from our data in the nondiabetic group of animals. We observe that long-term oral canagliflozin administration significantly reduces myocardial infarct size in the nondiabetic ZL rat heart. These data have 3 provocative implications:

1. The potentially paradigm-shifting observation that SGLT2 inhibitors may be repurposed for the management of high-risk nondiabetic patients with significant pre-existing cardiovascular disease.
2. Canagliflozin is not a pure diabetic drug, and possesses pleiotropic effects that extend beyond purely lowering serum glucose.
3. The cardioprotective effect of canagliflozin is only manifest when administered orally over a period of weeks, which challenges current thinking in terms of mechanisms that appear to extend beyond a direct effect upon either the myocardium or kidney.

### ex-vivo canagliflozin fails to protect the nondiabetic rat heart

In contrast to the long-term oral administration, the short-term administration of canagliflozin, ex vivo, administered at a concentration of 10 μmol/l (equivalent to the circulating concentration in patients taking canagliflozin, 300 mg once daily [7]) throughout the perfusion protocol, failed to reduce infarct size. This concentration of canagliflozin is also equivalent to a rat steady-state circulating serum canagliflozin concentration from oral digestion of drug, and a concentration that is sufficient to inhibit both SGLT2 and SGLT1, but insufficient to abrogate GLUT (glucose transporter) activity (12). The observation that short-term ex vivo administration of canagliflozin fails to protect the isolated heart may provide some further clues to the potential mechanism of action, because it appears to preclude a direct-acting cardioprotective effect of the drug upon the myocardium. Administering the drug ex vivo removes any confounding endocrine effects that the drug might elicit from any other organ system in vivo, as might occur in our long-term administration model. Thus, in the absence of infarct attenuation from ex vivo administration of canagliflozin, it would appear that the cardioprotective effect of SGLT inhibition is unlikely to be through the drug acting directly upon the myocardium itself and hints toward an endocrine (and downstream signaling) or metabolic effect to explain the beneficial effect of long-term oral administration of canagliflozin. However, our data appear not to support a metabolic effect: in our long-term canagliflozin model, the protection was seen ex vivo with a sole metabolic substrate: glucose at a concentration of 11 mmol/l. This makes preferential energy-substrate switching, as proposed in the ketone hypothesis (10), unlikely as an explanation for the cardioprotection observed. Following explantation and Langendorff perfusion of the heart, ketones will be rapidly washed out of the coronary circulation because the crystalloid-perfused Langendorff model is associated with far higher coronary flows than found in vivo (13). Thus, ketones will rapidly fall to negligible levels within the myocardium, and are unlikely to supplant the plentiful supply of glucose as the heart’s primary fuel source in the Langendorff perfused model. Of course, we have not excluded the role of endogenous myocardial glycogenesis, but interestingly, long-term SGLT2 inhibition leads to diminution of kidney and liver glycogen stores (14). The role of glycogen in myocardial ischemia reperfusion injury is complex—canonical succinate synthesis through gluconeogenesis during myocardial ischemia is likely beneficial, but potentially deleterious during reperfusion through reversal of complex II of the mitochondrial transport chain (15). The impact of glycogen depletion on myocardial injury would be interesting to study further.

The sodium hydrogen exchange (NHE) hypothesis appeared to be a strong and attractive contender to explain the cardioprotection in our long-term canagliflozin administration studies 16, 17. Previous investigations using cariporide and amiloride in animal models reveal highly efficacious anti-ischemic benefits of NHE inhibition against myocardial infarction, particularly when administered before the onset of myocardial ischemia 18, 19, 20, 21. Thus, we had anticipated the short-term ex vivo study to provide further evidence of infarct size limitation. Indeed, in the excellent study from Zuurbier’s group (17), with 3 μmol/l canagliflozin, they demonstrated highly effective attenuation of NHE activity. Given the similarity in concentration of canagliflozin in our and in Zuurbier’s cell-based model, we were surprised that we found no protection in our ex-vivo model. Might the protective effects of long-term administration of canagliflozin be mediated through NHE inhibition? Encouragingly, protection was observed in both diabetic and nondiabetic animals as expected. However, with 40 min of washout before induction of ischemia, it seems somewhat unlikely that significant quantities of canagliflozin would remain within the heart. Our data would therefore appear to suggest that the observed protection from long-term administration of canagliflozin is less likely to be mediated through NHE inhibition, but perhaps through another pleiotropic pathway capable of triggering a “memory” effect through activation of signaling cascades. Already identified candidate pathways include the aforementioned Jak/STAT3 pathway (9) that may also help attenuate oxidative stress and fibrotic myocardial remodeling (22) or perhaps through AMPK (23) (also found in kidney to reduce ischemia/reperfusion injury [24]), although these are not hypotheses that we have yet tested.

Finally, SGLT2 inhibitors have been found to imbue significant protection in the vasculature of diabetic ZDF rats, with preservation of endothelial function. This endothelial protection appears to be mediated through attenuation of long-term glucotoxicity and amelioration of oxidative stress (25). This could translate into myocardial protection ex vivo, but we did not find significant differences in coronary flow in our model between canagliflozin-treated versus control-treated animals (data shown in the Supplemental Appendix). Moreover, if the protection were mediated primarily as a mechanism designed to abrogate glucotoxicity, this hypothesis fails to explain why canagliflozin protects the nondiabetic heart. However, it would be interesting to repeat these experiments in the nondiabetic ZL rat to see whether the cytoprotective phenotype is evident in the absence of injurious elevated blood glucose.

### Canagliflozin-mediated cardioprotection appears independent of circulating glucose

As expected, we found canagliflozin to be highly effective at reducing circulating blood glucose in our diabetic rat model. Although we did not see the random blood glucose level in canagliflozin-treated diabetic ZDF rats fall into the nondiabetic range, the drug was nonetheless still highly effective at reducing infarct size, suggesting that complete restoration of random blood glucose into the “normal” nondiabetic range is unnecessary to imbue the cardioprotection observed. Moreover, canagliflozin failed to have an impact on circulating blood glucose levels in the nondiabetic animals: random glucose levels were identical in both nondiabetic control and canagliflozin-treated rats. There are 2 observations in respect to this data: 1) that canagliflozin can be administered to nondiabetic animals without fear of triggering potentially injurious hypoglycemia; and 2) that lowering blood glucose is not a prerequisite for attenuation of myocardial infarct size. Therefore, glucose lowering in the diabetic ZDF animals is a good biomarker of canagliflozin-mediated SGLT2 inhibition, but the in vivo lowering of glucose is not conditional for the triggering of infarct-size reduction when the heart is explanted and perfused ex vivo. Furthermore, as alluded to above, as the hearts were maintained with a perfused glucose concentration of 11 mmol/l throughout perfusion, any confounding effect of differences in circulating glucose concentration is effectively removed from our experiment.

Finally, it is also interesting to observe that long-term oral canagliflozin is equally protective in both nondiabetic and diabetic animals. This contrasts with the majority of cardioprotective interventions whose efficacy is blunted in the presence of the diabetic phenotype (11). This, therefore, leads us to speculate that the mechanisms of protection are different from, and potentially additive to, more established experimental models of myocardial protection, such as ischemic or pharmacological conditioning. If this were to be the case, then it offers the opportunity to augment myocardial protection through combined therapeutic approaches at the time of presentation of an acute coronary syndrome, to optimize patient outcome.

### Absence of renopreservation

In establishing our diabetic model, we wanted to determine the severity of the diabetic phenotype. The SGLT2 outcome studies have all been performed in models of established type 2 diabetes mellitus, and typically in patients with high cardiovascular risk. We therefore wanted to ascertain whether our model displayed characteristics of diabetic end-organ damage in the form of albuminuria. Our diabetic ZDF rats did indeed display evidence of significant albuminuria at the point at which the hearts were harvested for ex vivo Langendorff perfusion. The lack of any meaningful difference between the canagliflozin-fed and control ZDF rats is not, however, unexpected. The renoprotective effects of SGLT2 inhibition typically take many months to manifest 2, 26, which contrasts with the comparatively rapid separation of the cardiovascular outcome curves. We designed our study primarily as an investigation into cardioprotection; a study with renoprotection as a primary endpoint would likely mandate a much longer duration of drug treatment.

### Diabetic complications

It was initially surprising that the only serious, life-threatening complication found during our long-term study was infective. As might have been anticipated, the source of infection was, in both cases, urinary tract. However, these 2 events were in the nontreated control diabetic ZDF rats and not in animals treated with canagliflozin. In total, 2 animals in the control ZDF group had to be euthanized for serious sepsis; neither of the canagliflozin-treated groups (diabetic or nondiabetic) had evidence of septic complications. Both diabetic ZDF groups had significant glycosuria, whereas the untreated control ZDF also had significant hyperglycemia. The sepsis, therefore, is much more likely to be secondary to the uncontrolled diabetes in the control animals, whereas the infective risk associated with canagliflozin-induced glucosuria was easily managed by simple animal husbandry and hygiene methods. No animal deaths were found related to cardiovascular causes, but our study was not powered for this endpoint, nor was it run for a sufficient period for such complications to become manifest.

### Study limitations

In designing our studies, we accepted a number of compromises. To avoid the confusion that may ensue with polypharmacy, we did not treat the control diabetic animals to manage their hyperglycemia. These animals displayed high levels of glycemia, and 2 animals had septic complications that were rapidly identified and managed. We therefore feel that prolonging the duration of study beyond 4 weeks as designed would not have been feasible. However, the infarct size data are compelling: administering canagliflozin, irrespective of diabetic status, resulted in a pronounced reduction of myocardial infarct size.

As all diabetic patients in the clinical outcome studies were undertaken in the presence of antihyperglycemic agents, a future study may be constructed at the outset to include diabetic animals managed with metformin, the backbone of contemporary type 2 diabetic management. Indeed, this may well be mandated in any future study designed to look at cardiovascular complications and renal outcomes where much longer treatment periods would need to be considered.

We do not believe that the severity of the diabetes had an adverse impact upon the outcome of our study; in fact, the infarct size of the diabetic animals was entirely in line with previous short-term studies in other diabetic models (such as streptozocin-treated or Goto-Kakizaki lean diabetic rats) and from our own group and others 27, 28. However, having established that canagliflozin is cardioprotective, it would be useful to demonstrate that this protective phenotype is reproducible on top of existing strategies for managing elevated blood sugar.

Interestingly, it is well recognized that diabetic hearts, when Langendorff-perfused with glucose as the sole substrate, will have a smaller infarct size compared with the nondiabetic heart under the same conditions (see review [8]). Although a reductionist approach in metabolic substrate provision has its limitations, there are advantages in that we have excluded other potential metabolic substrates that have been postulated (such as ketone bodies). From our data, future more in-depth analysis of the myocardial metabolome may be undertaken, and for example, the impact of any glycogen depletion that may result from long-term SGLT2 inhibition, investigated.

Finally, our short-term canagliflozin study was performed in Sprague Dawley rats, rather than the ZL strain. Neither strain of rat are diabetic. Both strains reveal similar infarct sizes when subjected to 35 min of regional ischemia and 2 h reperfusion. Although there are differences between individual strains of murine and rat models, and their sensitivity to myocardial ischemia/reperfusion injury, given baseline similarities in infarct size, we would have expected canagliflozin to be as protective in Sprague Dawley rats as the ZL. The absence of protection observed is, therefore, informative, but minor strain differences cannot be completely excluded.

## Conclusions

We demonstrate that long-term oral administration of canagliflozin results in significant reduction in myocardial infarct size, irrespective of glucose lowering or the presence of diabetes. This protection appears not to be mediated via a direct effect of canagliflozin upon the myocardium, but via an intermediate signaling mechanism that has yet to be identified. Our study, therefore, provides new insights into the potential cardiovascular benefits of SGLT2 inhibition and even points to a potential and important translational repurposing of these drugs to reduce cardiovascular mortality in nondiabetic patients.

Perspectives **COMPETENCY IN MEDICAL KNOWLEDGE:** SGLT2 inhibitors are known to improve cardiovascular outcomes in high-risk diabetic patients. We demonstrate for the first time that SGLT2 inhibitors attenuate infarct size in both diabetic *and* nondiabetic rats. This class of antihyperglycemic drug, therefore, appears to have cardioprotective properties that extend beyond their ability to lower circulating blood glucose.

**TRANSLATIONAL OUTLOOK 1:** Long-term SGLT2 inhibition is cardioprotective, reducing myocardial infarct size following injurious myocardial ischemia. This is a favorable characteristic for a diabetic therapy, supporting their use in diabetic patients with high risk of, or established, cardiovascular disease.

**TRANSLATIONAL OUTLOOK 2:** Our data suggest that infarct limitation is also seen in nondiabetic animals, raising the tantalizing potential for repurposing these drugs to improve cardiovascular outcomes in all high-risk cardiovascular patients, irrespective of diabetic status.
