# Supplementary material for: SGLT2 Inhibitor, Canagliflozin, Attenuates Myocardial Infarction in the Diabetic and Nondiabetic Heart
Source: JACC Basic Transl Sci. 2019 Jan 30;4(1):15–26. doi: 10.1016/j.jacbts.2018.10.002 (PMC6390729; doi:10.1016/j.jacbts.2018.10.002)
Supplement: Supplemental Data [file mmc1.pdf]

Supplementary materials

1. Methods

All animal experiments were conducted in accordance to the United Kingdom Animals (Scientific Procedures) Act 1986 Amendment Regulations 2012. The protocols were as authorized in the project license PPL 70/8556, approved by the Animal Welfare and Ethical Review Board of UCL and Home Office.

1.1. Chronic administration of Canagliflozin prior to ex vivo experiments

1.1.1. Experimental Animals

The diabetes animal model used was the Zucker Diabetic Fatty *fa/fa* (ZDF) rat (ZDF-*Lepr*^{fa}/Crl). Male ZDF rats that are homozygous for the *fa* gene mutation of the leptin receptor gene (*fa* gene) [1] which leads to the development of obesity and insulin resistance at an early age (before 7 weeks of age) [2]. When fed on the Purina # 5008 Diet, the rats progressively develop hyperglycaemia associated with impaired pancreatic β-cell function, loss of pancreatic β-cell mass and decreased responsiveness of liver and extrahepatic tissues to the actions of insulin and glucose [2]. As these features are similar to type 2 diabetes in obese humans, the ZDF rat is a popular obese, type 2 diabetes model. The control rats were male Zucker Lean Fa/fa (ZL) rats (ZDF-*Lepr*^{fa}/+/Crl). ZL rats are heterozygous for the *fa* gene mutation and do not develop insulin resistance and diabetes. All ZDF and ZL rats were obtained from Charles River Laboratories (France).

1.1.2. Treatment Groups

The animals were housed in groups of two or three; and were provided diet and water *ad libitum* throughout the duration of the study. The animals were randomly assigned to four groups as follows:

1) Zucker Lean rats fed with a vehicle diet (ZL control group)

2) Zucker Lean rats fed with canagliflozin (ZL treatment group)

3) Zucker Diabetic Fatty rats fed with a vehicle diet (ZDF control group)

4) Zucker Diabetic Fatty rats fed with canagliflozin (ZDF treatment group)

All groups were placed on their allocated diet for 4 weeks duration. The diets were obtained from Research Diets, Inc (USA) and Table 1 describes the diet for each of the groups:

Group	Diet description
ZL control group<br>(n =9)	Laboratory Rodent Diet Purina Chow 5001* (LabDiet)<br>The composition of calories provided by the 5001* diet:<br>- protein 30%<br>- fat 13%<br>- carbohydrates 57%
ZL treatment group (n=10)	5001* chow incorporated with Canagliflozin 166.7mg/kg
ZDF control group<br>(n=8)	Formulab Diet Purina Chow 5008* (LabDiet)<br>The composition of calories provided by the 5008* diet:<br>- protein 27%<br>- fat 17%<br>- carbohydrates 56%
ZDF treatment group (n=9)	5008* chow incorporated with Canagliflozin 100mg/kg

Table 1. Diet description for each group

ZDF rats were fed with the Purina 5008* instead of 5001* as recommended by Charles River Laboratories to induce the consistent development of type 2 diabetes [3]. As ZDF rats have a higher daily food intake compared to ZL rats [4], the concentration of canagliflozin in the diet for ZDF treatment group was made to be lower than that for the ZL treatment group to allow for the plasma concentration of canagliflozin in both groups to be as similar as possible. From pharmacokinetic data provided to us from Jansen, the expected circulating concentration from the daily feed was 5 – 10 μmol/L, equivalent to the circulating concentration in man following oral administration of 300mg daily of Canagliflozin.

The rats weighed between 290-500g and were between 4 to 5.5 months of age on the day of the ex-vivo experiments. The variability in the body weights could not be avoided owing to differences in the strain.

1.1.3. Anthropometric measurements

As part of our model characterisation, the following parameters were measured on the day of the ex-vivo experiments:

Body mass index (BMI): BMI was determined by dividing the body weight (g) and the square of the nose tip to rump length (cm) [5].

1.1.4. Blood glucose measurements

Blood glucose measurements were taken on a weekly basis. Random blood glucose measurements were obtained by venous sampling from the rat tail vein and tested using a point-of-care blood glucose meter, the Accu-Chek Mobile blood glucose meter (Roche), which had been independently tested against International accuracy standards (ISO 15197:2013)[6]. On the day of the ex-vivo experiments, a further tail vein blood glucose sampling was carried out in awake rats.

1.1.5. Renal parameters

Blood urea nitrogen (BUN) measurement: Upon excision of the heart, whole blood was aspirated (between 3-5mL) and centrifuged at 5000rpm at 4°C for 15 minutes (eppendorf centrifuge 5804R v 8.4) to obtain serum. BUN was assessed using a commercially available assay kit (BioAssay Systems, Hayward, CA) validated in rodents[7].

Urine albumin/creatinine ratio measurement: After excision of the heart, urine was aspirated directly from the bladder using a needle and 1mL syringe. Unfortunately, urine extraction was only feasible in animals with full bladders, and subsequently analysis shown is for those animals where urine was collected. Albumin concentrations were measured by enzyme-linked immunosorbent assay (Bethly Laboratories, Montgomery, TX) and creatinine concentrations were assessed by a commercially available assay (Cusabio, Newark, DE)[8].

Urine dipstick measurement for glucosuria: About 0.1-0.3mL of the urine aspirated from the bladder was applied to a urine test strip to semi-quantitatively measure the extent of glucosuria (Mission Urinalysis Reagent Strips, ACON Laboratories Inc, San Diego, Ca, USA). The degree of glucosuria was expressed in one of six categories: negative (-), 5mmol/L (±), 15mmol/L (+), 30mmol/L (++), 60mmol/L (+++) and ≥110mmol/L (++++).

1.1.6. Isolated perfused heart experiment protocol (Langendorff model)

The rats were anaesthetised via intraperitoneal injection of pentobarbitone sodium solution (20%w/v, Animal Care UK, York, UK) at a final dose of 0.2-0.4g/Kg bodyweight. Anticoagulant heparin sodium (Rockhardt UK Ltd, Wrexham, UK) at a dose of up to 5000 units per Kg bodyweight was co-administered with the anaesthetic. Upon the onset of deep anaesthesia, identified as the loss of the pedal pain withdrawal reflex, slowing of heart rate and breathing, the hearts were excised and immediately secured onto the cannula of a gravity-driven Langendorff perfusion apparatus[9,10,11]. This enables a constant pressure perfusion system where the heart is cannulated via the aorta and retrogradely perfused and suspended within a warmed organ chamber. The perfusate consisted of the modified Krebs-Henseleit buffer containing NaCl 118.5 mM, $NaHCO_3$ 25.0 mM, d-glucose 11.0 mM, KCl 4.7 mM, $MgSO_4$ 1.2 mM, KH_2PO_4 1.2 mM and $CaCl_2$ 1.8 mM (Sigma Aldrich, Poole, UK), bubbled with 5% CO_2 and 95% O_2 [10,11].

The hearts were perfused at a constant pressure of 70-80mmHg, recorded using a pressure transducer. The temperature of the hearts were maintained between 36-38°C and monitored using a fine thermocouple (TM Electronics (UK) LTD, Goring by Sea, UK) retrogradely passed into the right ventricular outflow tract via the pulmonary artery. The heart rate, left ventricular end diastolic pressure (LVEDP) and left ventricular developed pressure (LVDP) were monitored via a fluid-filled intra-ventricular balloon inserted gently into the left ventricle via the left atrium. The changes in the parameters were recorded using the pressure transducer attached to the balloon. The perfusion pressure, temperature, heart rate, LVEDP and LVDP were recorded using a data acquisition hardware (PowerLab/8 SP by AD Instruments, Oxford, UK) and the readings displayed using the LabChart software (v7.3.8, AD Instruments, Oxford, UK). The coronary flow rate was measured via the collection of coronary effluent from the pulmonary artery. These physiological measurements were taken at regular intervals to ensure that the hearts met certain functional requirements with reference to pre-determined exclusion criteria (see Appendix 1).

After the setting up of the aforementioned physiological monitoring, external cardiac pacing was delivered to the heart via the right atrium at a constant rate of 300 beats per minute to simulate physiological cardiac electrical conduction. The pacing system consisted of a pulse

generator, a pacing lead (positive pole), which was placed in the right atrium, and a crocodile clip (negative pole), which was attached to the aortic cannula. Cardiac pacing was delivered at an energy output of 1V and a pulse width of 10ms. Cardiac pacing was used throughout the experimental protocol, which consisted of a period of stabilisation for 40 minutes, regional ischaemia by occlusion of the left anterior descending artery (LAD) for 35 minutes and reperfusion for 120 minutes.

1.2. Acute administration of Canagliflozin during the ex vivo experiments

1.2.1. Experimental Animals

Male Sprague-Dawley rats (weight range between 290-350g) were used as the non-diabetic rat model.

1.2.2. Baseline characteristics

The same anthropometric, blood glucose and renal data were obtained as described in the first study.

1.2.3. Isolated perfused heart experiment protocol (Langendorff model)

The rats were anaesthetised and the hearts were excised and immediately secured onto the cannula of a gravity-driven Langendorff perfusion apparatus in the same method as per the first study. The protocol applied was similar to the first study which was a period of stabilisation for 40 minutes, regional ischaemia by occlusion of the left anterior descending artery (LAD) for 35 minutes and reperfusion for 120 minutes.

1.2.4. Group allocation

The hearts were allocated into one of the two groups:

1) Control group (n = 6): Isolated heart perfused throughout the study with modified Krebs-Henseleit buffer (as described in first study) with the addition of 0.05% dimethyl sulfoxide (DMSO)
2) Canagliflozin group (n = 6): Isolated heart perfused throughout the study with modified Krebs-Henseleit buffer with the addition of 10µmol/L Canagliflozin dissolved in 0.05% DMSO

1.2.5. Myocardial infarct size determination

At the end of reperfusion for the experiments in the first and second studies, the suture around the LAD was tightened and Evans blue (0.25% in saline) was infused into the coronary system via the aortic cannula, thereby staining only the perfused (non-risk) myocardium blue. The hearts were then frozen at -20°C overnight and then sectioned transversely into five equal slices of 2mm perpendicular to the long axis and distal to the LAD ligation site. The heart sections were then immersed in 1% triphenyltetrazolium chloride (TTC) in phosphate buffer solution (PBS) at 37°C for 20 minutes. TTC is a viable dye that stains the viable tissue pink while the infarcted tissue remains unstained and pale. To improve the contrast between viable and non-viable tissue, the stained heart slices were then placed in formalin (40% formaldehyde in 0.9% NaCl) overnight. The heart slices were subsequently scanned and after computer planimetry (Image J 1.50i), the data were expressed as a ratio of infarct area to the area at risk. To check the consistency of the LAD ligation, data on the ratio of area at risk to total area were also obtained.

1.3. Statistical Analysis

1.3.1. Preliminary Experiments and Power Calculation

A randomized, blinded preliminary study was undertaken in un-paced diabetic (ZDF) and non-diabetic (ZL) rats (n=4 per group). The means of the controls (±standard error of the mean) were 37±7% and 38±13% for ZL and ZDF rats respectively. Mean of the Canagliflozin-treated animal hearts were 29±6% and 29±3% respectively. From these data, we estimate that Canagliflozin reduced infarct size by 25%, with a whole group standard deviation of 16%.

Based on the preliminary data, we calculated sample size: with power (1-β) of 80%, and setting significance (α) at 5%, 1-way ANOVA and 4 groups, the sample size was determined to be n=8 per group.

Note that we found that heart rate in Langendorff perfused diabetic hearts were significantly lower than in non-diabetic heart (see Supplemental Results, section 2.3) and subsequently for the main study, all hearts were paced. Interestingly, with pacing, we found that the inter-experiment standard deviation reduced, leading to an increase in statistical power observed.

2. Results

2.1. Diabetic and non-diabetic rat growth curves: Canagliflozin versus control

Zucker Lean (ZL) and Zucker Diabetic Fatty (ZDF) rats were bought in aged 3-4 months. Baseline weight characteristics are shown in figure 1 below. The non-diabetic ZL were significantly lighter (300g) than the diabetic ZDF rats (372g). At baseline, there were no significant differences between control and Canagliflozin-fed rats.

Figure S1: Weight growth curves – Non-diabetic Zucker Lean (ZL) versus diabetic Zucker Diabetic Fatty (ZDF) rats, control diet versus Canagliflozin-fortified diet.

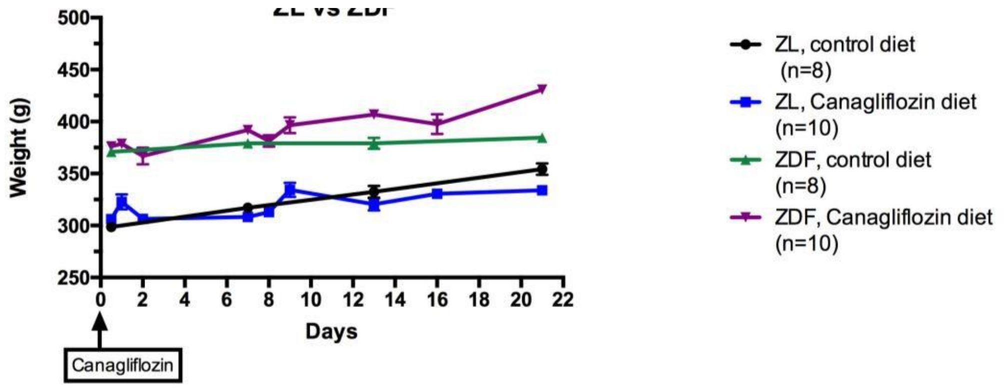

Days	0	1	2	7	8	9	13	16	21
ZL control	299 ± 3			317 ± 5			333 ± 6		354 ± 5
ZL Cana	306 ±5	323 ± 7	307 ± 3	308 ± 4	313 ± 0	3334 ± 7	321 ± 6	331 ± 3	334 ± 5
ZDF control	371 ± 2			379± 4			379± 6		385± 4
ZDF Cana	376 ± 3	379 ± 5	367 ± 8	392 ± 5	382 ± 6	397 ± 8	408 ± 4	398 ± 10	431 ± 1

All animals gained weight during the Canagliflozin/ control wash-in period. Control ZL rats increased 55g, from 299±3g to 354±5g. This weight gain was significantly blunted by the administration of Canagliflozin, with a mean weight gain of just 28g (306±5g to 334±5g). SGLT2 inhibition is associated with significant glucosuria (confirmed on urinalysis in our animals), and the consequent calorific loss is felt to be the most likely explanation for this difference – a change consistent with the adult human data from the EMPAREG and CANVAS studies.

Our diabetic animals behaved somewhat differently. Untreated diabetic ZDL rats gained, on average just 14g over the wash-in period. However, Canagliflozin enabled the ZDL rats to gain weight – on average, 55g (376±3g to 431±1g). At first glance, these data appear

contrary to the human data presented in the SGLT2 outcome studies. However, the diabetic control ZDL animals received no therapy to control glycaemia (see glucose profile, figure 1B, main paper). Thus, these rats are in a catabolic state, and their weight growth blunted. Placebo-control diabetic patients in the clinical outcome studies had standard-of-care diabetic control, with a minimum of metformin. Their glycaemic control therefore was substantially better than in our control rats, and moreover, the placebo-treated patients would also have received oral hypoglycaemics that may in fact have encouraged weight gain. SGLT2 inhibition was added to this background therapy, and the calorific loss through glucosuria encouraged weight loss in the treatment arms of the two outcome studies. By contrast, Canagliflozin mono-therapy in our diabetic rats improved diabetic control and were, therefore, in a better metabolic state than their control brethren – thus enabling weight gain on a pair to that seen in the non-diabetic control ZL rats.

2.2. Representative heart slices

Infarct size data are summarized in figures 4 and 5, main paper. Figure S2 shows representative heart slices from these experiments.

Figure S2: Representative heart slices – (A) Chronic Canagliflozin administration versus control chow; (B) Acute, ex-vivo, Canagliflozin administration versus DMSO control

A: Chronic Canagliflozin administration versus standard Chow

Zucker Lean, non-diabetic, vehicle control diet

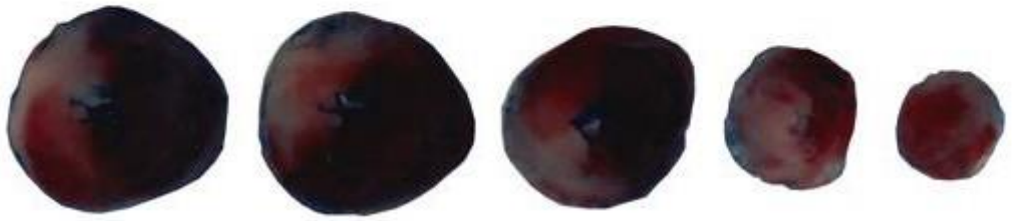

Zucker Lean, non-diabetic, Canagliflozin diet

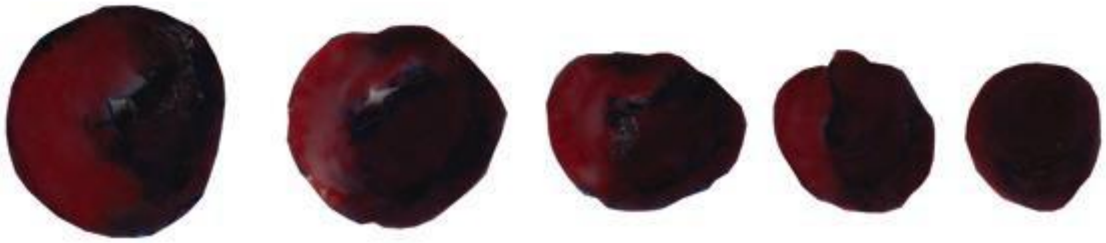

Zucker Diabetic Fatty, diabetic, vehicle control diet

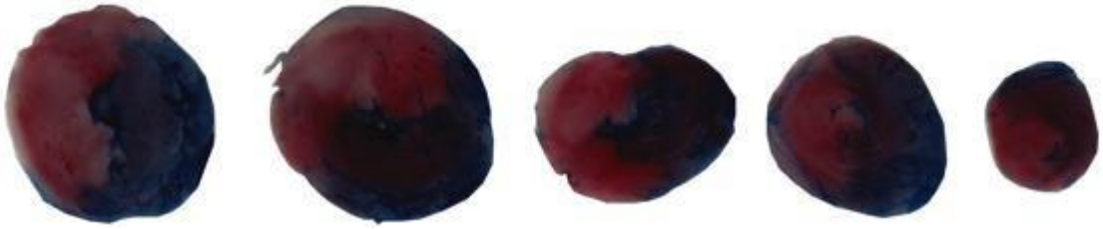

Zucker Diabetic Fatty, diabetic, Canagliflozin diet

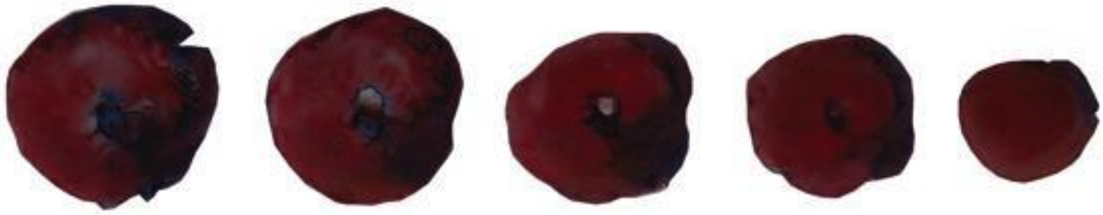

B: Acute, ex-vivo, administration of Canagliflozin in non-diabetic Sprague-Dawley rat

Vehicle (DMSO) control

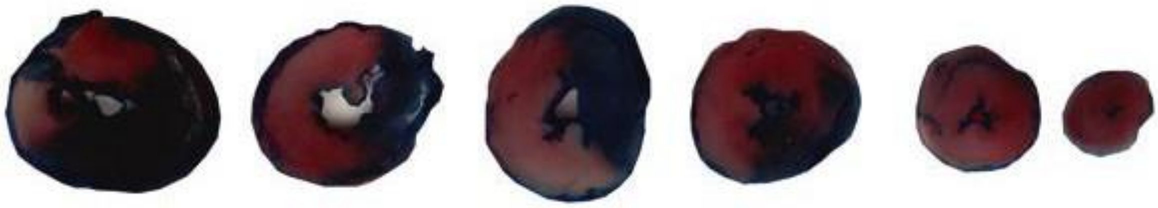

Canagliflozin

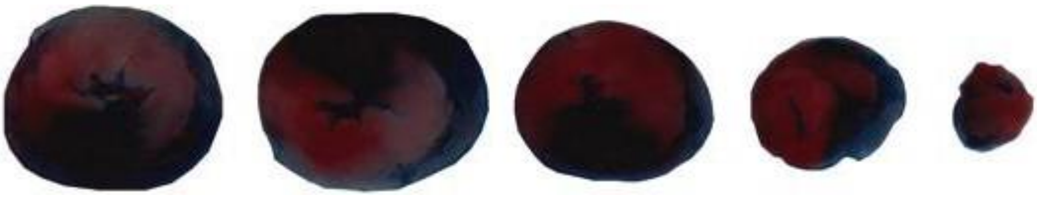

2.3. Preliminary data: heart rate is significantly lower in diabetic rat heart

Preliminary experiments were undertaken in un-paced, Langendorff-perfused hearts: vehicle-control or Canagliflozin-fortified diet pre-treatment is identical to the animals used in the main study.

Non-diabetic animals had a baseline heart rate of 232-241 beats per minute, but diabetic animals had a significantly lower heart rate of between 113-171 beats per minute (see figure S3 below). Moreover, the standard deviation of heart rate was also much higher in the diabetic animals. The combination of these observations led us to the decision to undertake the main study with cardiac pacing to remove the potential confounding effect of reduced heart rate and altered cardiac work.

Interestingly, Canagliflozin pre-treated hearts had a numerically higher mean heart rate compared to control ZDF rats (171±18 versus 113±28 bpm), but owing to the large variance of the data, this does not reach statistical significance.

Figure S3: Heart rates in isolated, ex-vivo perfused non-diabetic, Zucker Lean (ZL) and diabetic, Zucker Diabetic Fatty (ZDF) rat heart. Animals were pre-fed control, vehicle diet (Veh) or chow fortified with Canagliflozin (Cana).

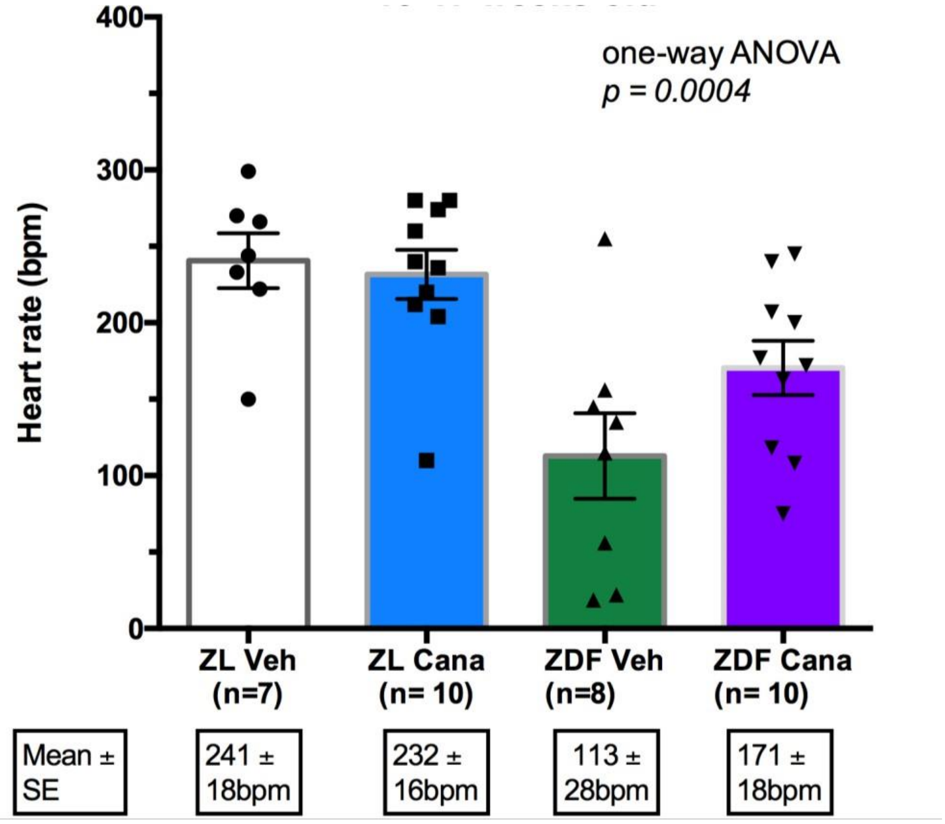

2.4. Anthropomorphological and macroscopic organ data

2.4.1. Liver morphology – Chronic administration of SGLT2 inhibitor in Zucker rat

The Zucker Diabetic Fatty (ZDF) rat was found to have a significantly larger liver to body weight ratio than the non-diabetic, Zucker Lean (ZL) rats. Prior administration of Canagliflozin had no impact upon the macroscopic or organ-weight:body-weight ratio (figure S4).

Figure S4: Liver weight to body weight ratio in non-diabetic and diabetic animals on control or Canagliflozin-fortified diet.

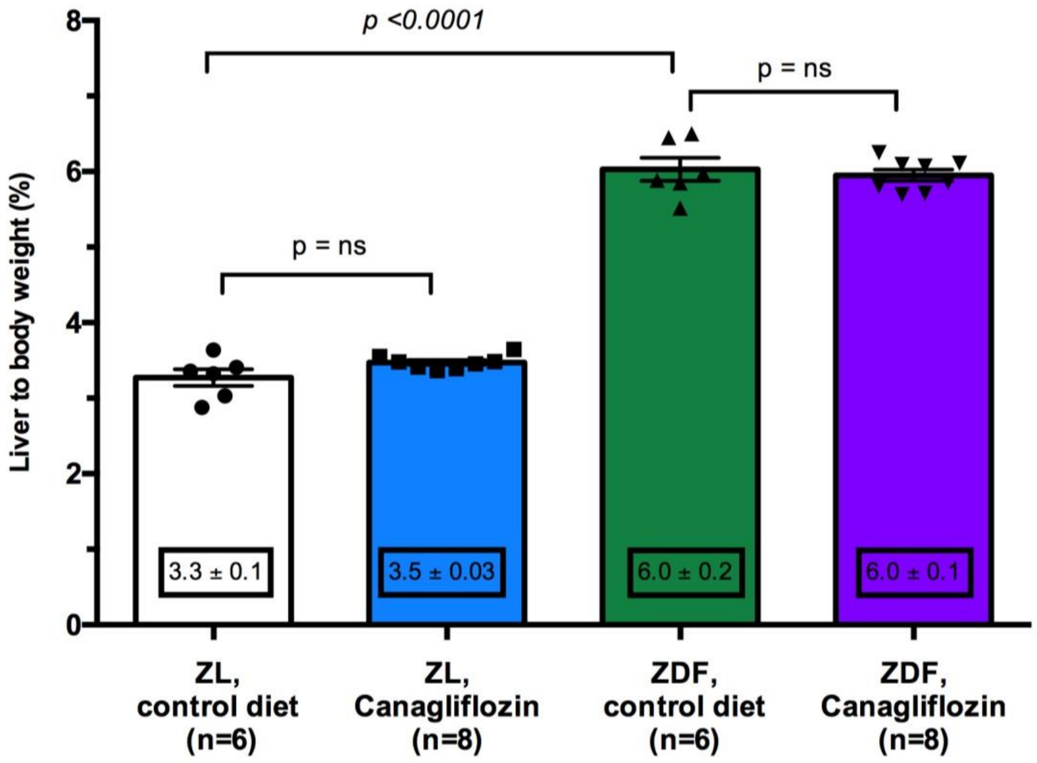

2.4.2. Summary of animal and morphological data from acute, ex-vivo study

Supplemental table R1: Baseline characteristics of the animals in the second study: Evaluating the effects of acute administration of Canagliflozin to isolated non-diabetic rat hearts

Characteristic	Control (n = 6)	Canagliflozin (n = 6)	p Value

Random blood glucose (mmol/L)	8.7 ± 0.3	8.2 ±0.2	0.16
Weight (g)	320 ± 7	317 ± 8	0.76
Body mass index (g/cm^2)	0.61 ± 0.02	0.61 ± 0.01	0.90
Waist to length ratio	0.74 ± 0.01	0.73 ± 0.01	0.42
Heart weight to tibial length ratio	0.33 ± 0.02	0.32 ± 0.01	0.52
Serum BUN (mg/dL)	7.8 ± 0.9	8.7 ± 1.1	0.54
Urine Albumin/Creatinine ratio (mg/g)	162 ± 29	136 ± 17	0.47
Cannulation time (s)	60 ± 7	54 ± 13	0.69
Heart rate at the end of stabilization (bpm)	266 ± 19	277 ± 12	0.63
Coronary flow rate at the end of stabilisation (mL/min)	11 ± 1	12 ± 1	0.35
LV Developed Pressure at the end of stabilisation (mmHg)	114 ± 8	111 ± 8	0.77

2.5. Functional data

In addition to infarct size, we also recorded functional parameters for the main study. All hearts were paced at 300bpm. Coronary flow and left ventricular pressure traces were recorded. These data are summarized in figure S5 and figure S6 below.

Figure S5: Chronic administration of Canagliflozin in diabetic ZDL and non-diabetic ZL rats. Canagliflozin had no significant impact upon coronary flow rate in non-diabetic ZL rat heart. Interestingly, there was a significantly higher coronary flow rate in the Canagliflozin-treated diabetic ZDF rat hearts compared to the untreated control (*p<0.05).

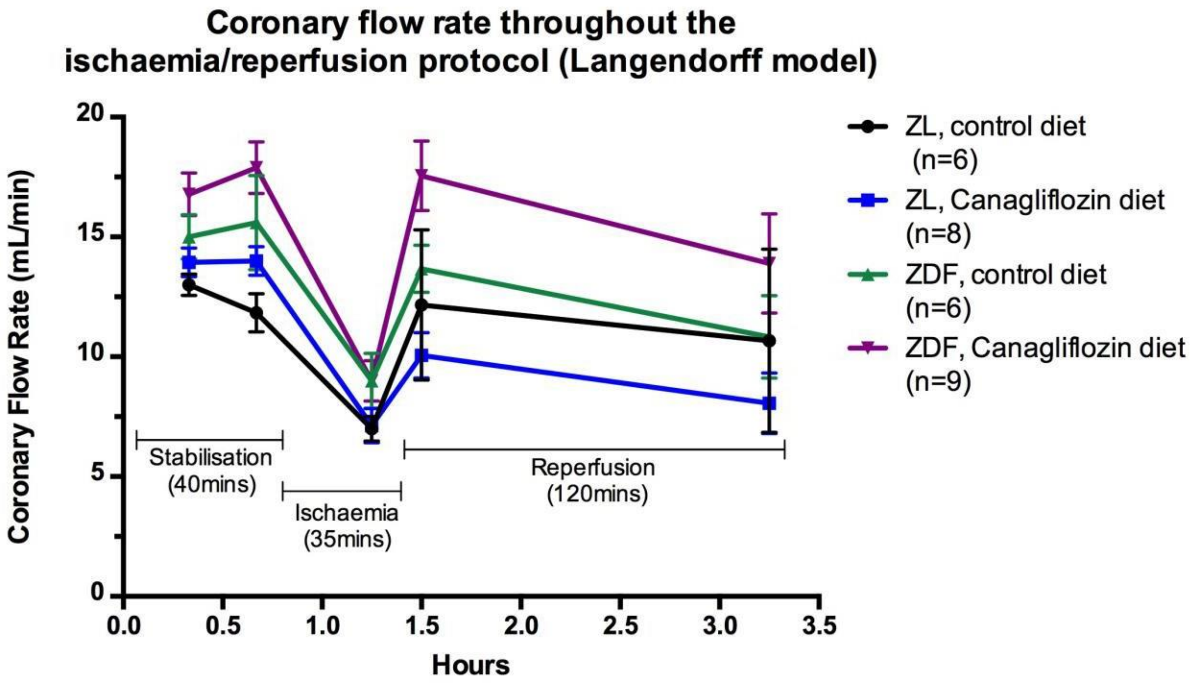

Canagliflozin resulted in improved coronary flow in the diabetic, ZDF rat hearts, suggesting that 4-week pre-treatment with Canagliflozin had a vascular protective effect consistent to that seen with SGLT2 inhibitors in thoracic aorta (see reference 25, main paper). However, a similar preservation in coronary flow was not observed in the non-diabetic rat, suggesting that the infarct-sparing properties of chronic Canagliflozin administration is not determinate upon coronary flow preservation.

Figure S6: Left ventricular developed pressure in Langendorff perfused hearts pre-treated with either control chow, or Canagliflozin fortified feed. There was numerically

higher left ventricular pressure in both non-diabetic and diabetic ZL and ZDF rat heart from animals pre-treated with Canagliflozin, in line with the reduction of infarct size. However this did not reach statistical significance.

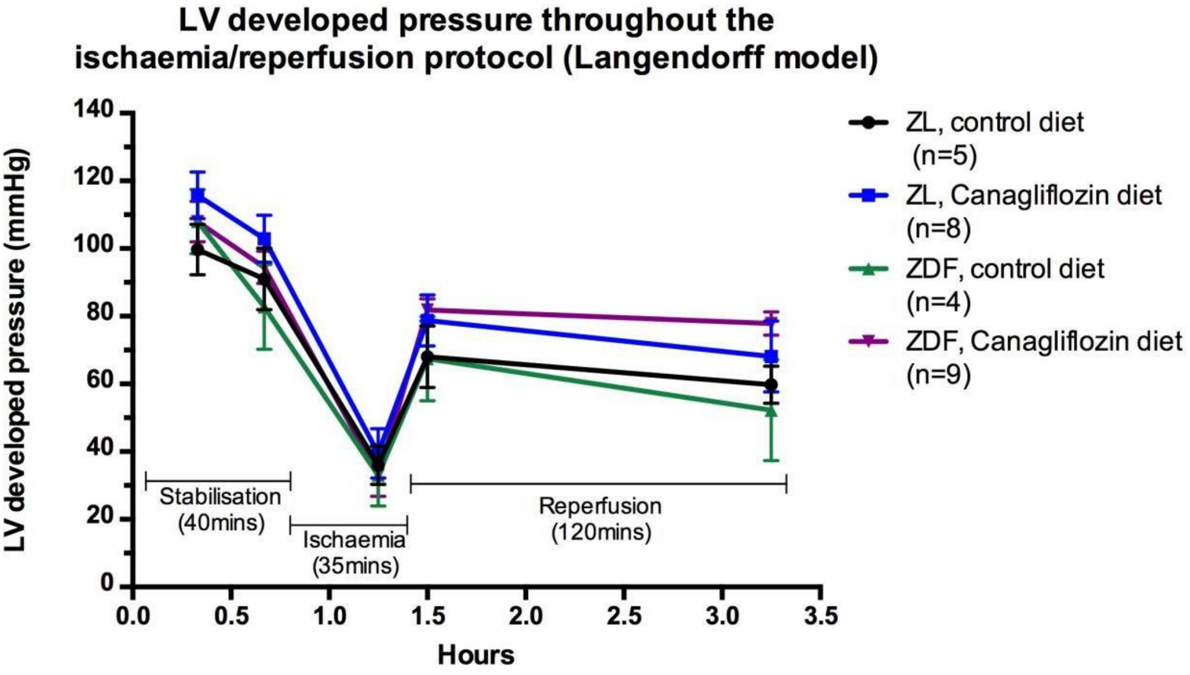

Interestingly, Left ventricular pressure recovery following injurious ischaemia/ reperfusion injury was numerically greater in the Canagliflozin-treated groups, consistent with the reduction of infarct size. However, these differences failed to reach statistical significance. This study was not powered to measure differences in LV developed pressure as an outcome, and some experiments suffered with intraventricular balloon failure, leading to exclusion of functional data in the above analysis.

3. References for Methods (Supplementary material):

1) Phillips MS, Liu Q, Hammond HA, Dugan V, Hey PJ, Caskey CJ, Hess JF. Leptin receptor missense mutation in the fatty Zucker rat. Nat Genet 1996; 13: 18-19.
2) Shiota M, Printz RL. Diabetes in Zucker Diabetic Fatty Rat. In: Joost HG, Al-Hasani H, Schürmann A, eds. Animal Models in Diabetes Research. Methods in Molecular Biology (Methods and Protocols). Totowa, NJ: Humana Press; 2012. vol 933.
3) Charles River. ZDF Rat Details. https://www.criver.com/products-services/find-
4) Alemzadeh R, Tushaus KM. Diazoxide Prevents Diabetes in ZDF Rats. Endocrinology 2004; 145(12): 5476–5484.

5) Novelli ELB, Diniz YS, Galhardi CM, Ebaid GMX, Rodrigues HG, Mani F, Fernandes AAH, Cicogna CH, Novelli JVLB. Anthropometrical parameters and markers of obesity in rats. Lab Anim 2007; 41: 111–119.
6) Freckmann G, Schmid C, Baumstark A, Pleus S, Link M, Haug C. System Accuracy Evaluation of 43 Blood Glucose Monitoring Systems for Self-Monitoring of Blood Glucose according to DIN EN ISO 15197. J Diabetes Sci Technol 2012; 6(5): 1060-1075.
7) Kolatsi-Joannou M, Price KL, Winyard PJ, Long DA. Modified citrus pectin reduces galectin-3 expression and disease severity in experimental acute kidney injury. PLoS ONE 2011; 6: e18683.
8) Long DA, Kolatsi-Joannou M, Price KL, Dessant-Baradez C, Huang JL, Papakrivopoulou E, Hubank M, Korstapie R, Gnudi L, Woolf AS. Albuminuria is associated with too few glomeruli and too much testosterone. Kidney Int 2013; 83 (6): 1118-1129.
9) Langendorff 0. Untersuchungen am uberlebenden saugethierherzen. Pfliigers Arch Ces Physiol 1895; 61: 291-332.
10) Bell RM, Mocanu MM, Yellon DM. Retrograde heart perfusion: the Langendorff technique of isolated heart perfusion. J Mol Cell Cardiol 2011; 50: 940-950.
11) Sutherland FJ, Hearse DJ. The isolated blood and perfusion fluid perfused heart. Pharmacol Res 2000; 41(6): 613–627.

4. Appendix 1 (Methods)

Summary of exclusion criteria for the Langendorff technique of isolated rat heart perfusion for the regional ischaemia protocol

Parameter	To exclude if
Time to perfusion (min)	> 3
Coronary flow at the end of stabilisation (ml/min)	< 9 or > 28
Change in coronary flow between stabilisation →ischaemia (ml/min)	<3
Heart rate (beats per min) during stabilisation	< 70 or > 400
Sustained arrhythmia duration during stabilisation (min)	>3
Left ventricular developed pressure (mm Hg) at the end of stabilisation	< 60
Temperature (°C) throughout protocol	<35.5 or >38.5 for >60s
Area at risk to total area (%)	<40 or >80
Adapted from: Bell RM, Mocanu MM, Yellon DM. Retrograde heart perfusion: the Langendorff technique of isolated heart perfusion. J Mol Cell Cardiol 2011; 50: 940-950.	
